# Body composition, bone mineral density, and functional impairment in axial spondyloarthritis: a 36-month longitudinal study

**DOI:** 10.1186/s12891-025-09088-8

**Published:** 2025-09-30

**Authors:** Chiara Ceolin, Sara Bindoli, Giacomo Cozzi, Benedetta di Marzio, Mariagrazia Lorenzin, Marina De Rui, Paolo Sfriso, Andrea Doria, Giuseppe Sergi, Roberta Ramonda

**Affiliations:** 1 Geriatrics Division, Padova University Hospital, Padova, Italy; 2 Department of Medicine (DIMED), Padova University Hospital, Padova, Italy; 3 Department of Neurobiology, Care Sciences and Society, Aging Research Center, Karolinska Institutet and Stockholm University, Stockholm, Sweden; 4 Rheumatology Unit, Padova University Hospital, Padova, Italy

**Keywords:** Axial spondyloarthritis, Sarcopenia, Older adults, BASDAI, BASFI, Bone mineral density

## Abstract

**Background:**

Axial spondyloarthritis (ax-SpA) is a chronic inflammatory disease affecting the axial skeleton, peripheral joints, And entheses, often leading to pain, stiffness, And extra-articular complications. Its impact on muscle and bone health has gained attention, as chronic inflammation, reduced physical activity, sedentary behaviour, and glucocorticoid therapy, may alter body composition, particularly lean mass and adipose tissue distribution. This study explores the association between disease activity, body composition, and bone parameters in ax-SpA patients over a 36-month period.

**Methods:**

This longitudinal study was conducted at Padua University Hospital (Italy). The following data was collected at baseline And 36 months: medical history, phospho-calcium metabolism, anthropometric measurements, handgrip strength (using hand dynamometer), sit-to-stand test, and Dual-Energy X-ray Absorptiometry assessments. Patients completed Bath Ankylosing Spondylitis Functional Index (BASFI), Bath Ankylosing Spondylitis Disease Activity Index (BASDAI), and Health Assessment Questionnaire (HAQ). Correlations between questionnaire scores and clinical variables were analysed, and changes from baseline to follow-up were assessed using paired comparisons.

**Results:**

Twenty participants (10 ax-SpA, 10 matched controls) were enrolled. At baseline, no significant differences in bone and body composition were found between groups. In ax-SpA patients, BASFI correlated with BMI (*r* = 0.800, *p* < 0.01), fat percentage (*r* = 0.808, *p* < 0.01), and fat mass index (*r* = 0.903, *p* < 0.01), while BASDAI correlated with sit-to-stand performance (*r* = 0.677, *p* < 0.05) and fat percentage (*r* = 0.700, *p* < 0.05). After 36 months, significant improvements were observed in sit-to-stand scores [from 17.37 (7.47) to 11.98 (3.81), *p* = 0.02] and femoral neck BMD [from 0.89 (0.13) to 1.02 (0.14), *p* = 0.01]. Sit-to-stand improvements correlated with BASFI (*r* = 0.78, *p* < 0.01), and ASMMI changes correlated with HAQ (*r* = 0.92, *p* < 0.001).

**Conclusion:**

Our findings suggest that greater muscle mass and physical performance are associated with lower disease activity and improved quality of life in ax-SpA patients. These associations support integrating pharmacologic treatment with structured exercise, although causal inferences cannot be drawn from this observational design. Further studies are needed to clarify the directionality and underlying mechanisms.

**Trial registration:**

Not applicable.

**Supplementary Information:**

The online version contains supplementary material available at 10.1186/s12891-025-09088-8.

## Background

Spondyloarthritis (SpA) is the second most prevalent chronic rheumatic disease (RD) in both sexes [1]. Axial (ax)-SpA can affect the spine, as well as entheses and joints, resulting in structural damage and new bone formation in the axial and peripheral skeleton. The disease may present both in a radiographic form (r-axSpA) with structural changes of the sacroiliac (SI) joint, according to the modified New York criteria [2], and a non-radiographic form (nr-axSpA), characterised by the absence of X-ray findings on the spine, entheses And joints. The worldwide prevalence is about 1% and the disease with the usual disease onset < 45 years old [3]. The hallmark early symptoms include inflammatory back pain and stiffness, which tend to worsen with inactivity [4]. Furthermore, the disease is often associated with extra-articular manifestations, including uveitis, psoriasis, and chronic inflammatory bowel disease, further complicating its clinical presentation [4]. Chronic pain and progressive joint dysfunction frequently result in a more sedentary lifestyle, which is further exacerbated during acute inflammatory flares [4, 5].

Beyond its well-documented complications—such as cardiovascular disease, osteoporosis and fractures, gastrointestinal disorders, and pulmonary involvement [6]—there has been growing interest as to the potential impact of SpA on muscle and bone health. Furthermore, recent years have seen considerable debate on whether sarcopenia may represent a complication associated with SpA [7]. According to the European Working Group on Sarcopenia in Older People (EWGSOP), sarcopenia is defined as an abnormal reduction in muscle mass associated with decreased skeletal muscle strength and/or impaired physical performance, which increases the risk of disability and reduced QoL [8]. While primary sarcopenia is mainly linked to age-related muscle decline [9], the chronic inflammatory nature of SpA suggests a potential association with secondary sarcopenia, particularly in patients with high disease activity. However, current evidence remains inconclusive regarding the prevalence of sarcopenia in SpA [10]. Several studies have reported muscle impairment in SpA, particularly in terms of reduced strength and power [10]. Although the precise mechanisms underlying this phenomenon remain a matter of debate, multiple factors are likely involved, including chronic inflammation, decreased physical activity, sedentary behaviour, glucocorticoid therapy, and neuromuscular impairment. Among these, inflammation plays a crucial role, potentially contributing to the onset of sarcopenia, further impairing muscle function, and worsening of disease burden [11].

Despite growing interest in this topic, available evidence remains limited. A major challenge in the existing literature is the heterogeneity in assessing muscle health, the diversity of studied populations, and the lack of longitudinal data. Given these considerations, it has been hypothesised that disease severity may be linked to changes in body composition, particularly regarding the distribution of lean mass and adipose tissue. It appears that higher disease activity might be associated with increased adipose tissue, which is known to contribute to pro-inflammatory cytokine production, and a concomitant reduction in muscle mass. However, to date, only a limited number of studies have explored the relationship between disease activity indices, such as the Bath Ankylosing Spondylitis Disease Activity Index (BASDAI) and the Bath Ankylosing Spondylitis Functional Index (BASFI), and body composition, with most investigations being cross-sectional and thus lacking longitudinal insights.

Against this background, our study is the first to evaluate, over a 36-month follow-up period, the progression of ax-SpA in patients naïve to biologic therapy or with bio-failure requiring a treatment switch, in relation to changes in body composition. Our aim was to explore the relationships between disease activity questionnaires, bone parameters, and body composition by analysing data collected both at baseline and after treatment initiation or modification.

## Materials and methods

### Study population

Patients were recruited from the Rheumatology Unit, Department of Medicine at the University Hospital of Padua, while healthy volunteers were selected from the medical and university staff in Padua through a dedicated recruitment call. The inclusion criteria for the study included individuals with ax-SpA (ASAS criteria), aged over 18 years, who had either received a new prescription for biologic drugs or experienced bio failure requiring a treatment change.

The study protocol has been approved by the Local Ethics Committee (Ethics Committee for Clinical Experimentation of the Province of Padua, (number CESC:3024P/AO2014) and in compliance with the principles of the Declaration of Helsinki. Each enrolled subject provided written informed consent to participate in the research.

### Data collection

At baseline And after 36-month of follow-up, each participant underwent comprehensive data collection, including:

- *Patient characteristics*: Detailed physiological, clinical, and pharmacological information was collected for each participant. Adequate calcium intake was verified through dietary interviews conducted by clinical staff, while vitamin D supplementation was recorded from medical charts and patient reports.
- *Laboratory data*: Fasting blood samples were collected in the early morning to enable the analysis of various parameters, such as serum calcium, phosphorus, parathyroid hormone (PTH), And 25-hydroxy-vitamin D (25-OH-D). All laboratory analyses were conducted following standardised procedures at the Laboratory Medicine Unit of the University Hospital of Padua.
- *Anthropometry*. Calf circumference was measured around the maximum circumference in the middle of the calf length. Mid–upper arm circumference was measured in the right upper arm at the midpoint between the tip of the shoulder and the tip of the olecranon process.
- *Physical Performance measures.* Gait Speed: Walking speed was assessed by timing participants over a 4-meter distance at their usual pace. The best performance from two trials was recorded in meters per second. Participants could use canes or walkers if necessary.
- *Muscle Strength: Handgrip test*: Upper limb strength was evaluated using DynEx electronic hand dynamometers (MD Systems, Westerville, OH, USA) by trained medical personnel. Participants performed three trials for each hand during the handgrip strength test. The maximum value from each hand was recorded, and the average of the dominant and non-dominant hand measurements was calculated. *Chair Stand Test*: Participants were instructed to stand up and sit down five times as quickly as possible with their arms crossed over their chest. The total time taken to complete the test, measured in seconds, was recorded.
- *Bone measurements*: we employed Dual Energy X-ray Absorptiometry (DXA) utilising fan-beam technology to measure bone mineral density (BMD) at the proximal femur (femoral neck and/or total hip) And lumbar spine for each patient. All DXA scans were performed using the same Hologic QDR 4500 W densitometer, operated by the same trained technician throughout the study. Calibration procedures, software version, and anatomical positioning protocols were kept constant at both baseline and follow-up to ensure measurement consistency and reliability. Total body DXA examination was carried out to measure Fat-free mass (FFM), Fat mass (FM), and Appendicular Skeletal Muscle Mass (ASMM). The Indices of Fat Mass (FM-Index) and Appendicular Skeletal Muscle Mass Index (ASMMI) were calculated dividing the FM and ASMM by the height in squared meters. *BASFI questionnaire*: BASFI is a measure of physical function [12, 13]. It is a patient-assessed, validated, composite index made up of 10 questions that address function and the patient’s ability to manage his or her ax-SpA.
- *BASDAI questionnaire*: BASDAI is one of the most used instrument to measure disease activity in axSpA [14]. It comprises six patient-reported questions relating to five major symptoms: fatigue, axial pain, peripheral pain, tendon and ligament inflammation and morning stiffness. The domain morning stiffness is calculated using the average of two separate questions about stiffness (level and duration).
- *Health Assessment Questionnaire (HAQ)* is a widely used tool designed to assess the functional status of individuals, particularly those with chronic conditions like Rheumatoid Arthritis. It measures a patient’s ability to perform daily activities, reflecting their functional limitations. The HAQ is valuable because it correlates functional status with long-term morbidity and mortality, providing insights into a patient’s QoL and the impact of their condition beyond physical symptoms [15].

All physical performance assessments were conducted by the same trained personnel at both baseline and follow-up to ensure consistency and reliability.

A control group of healthy volunteers was included at baseline to provide reference values and enable cross-sectional comparison with SpA patients, allowing for interpretation of potential disease-related alterations in body composition and function at study entry. Controls were individually matched to patients based on age (± 5 years), sex, and BMI (± 2 kg/m²).

### Statistical analysis

No formal sample size calculation was performed, as this was a pilot observational study designed to explore preliminary associations and inform future research.

Categorical variables are presented as counts and percentages, while continuous quantitative variables are expressed as mean (standard deviation, SD) or median (interquartile range, IQR), depending on distribution. The normality of continuous variables was assessed using the Shapiro–Wilk test. Comparisons between groups (patients and controls) were performed using the independent-samples t-test or the Mann–Whitney U test, as appropriate. Categorical variables were analysed using the Chi-square test. For within-group comparisons over time, paired t-tests or Wilcoxon signed-rank tests were used. Correlations were assessed using Pearson’s or Spearman’s coefficients depending on variable distribution.

Reduced muscle strength was defined as a handgrip test score of < 27 kg in men and < 16 kg in women, or a chair stand test time of > 15 s, according to international consensus guidelines [8]. Low muscle mass was defined as an ASMMI value of < 7.0 kg/m² in men and < 5.5 kg/m² in women [8], or as a calf circumference below the median.

A two-tailed *p*-value < 0.05 was considered statistically significant in all analyses. Statistical analyses were performed using SPSS software (version 29).

## Results

The sample consisted of 10 patients with SpA (males 90%, mean age ± SD 43.8 ± 11.5) And 10 controls (males 90%, mean 44.9 ± 16.5). The clinical and demographic characteristics of the patients included are displayed in Table 1. At the initial assessment, all subjects were treated with biological disease-modifying antirheumatic drugs (bDMARDs). Specifically, 9 patients (90%) received TNF-alpha inhibitors, and one patient (10%) received an IL-17 A inhibitor. Regarding Vitamin D supplementation, 50% of the subjects were receiving it. All participants had an adequate calcium intake, and no specific dietary regimens were recorded. None of the patients had a history of previous fractures in their medical history. Overall, the comparison between the two groups revealed no significant differences across the analysed parameters (Table 2). Both groups had a similar mean age (approximately 44 years) and an identical sex distribution (10% female). Strength measurements, including handgrip performance, and body composition parameters showed no meaningful differences. Six patients with SpA (60%) had reduced muscle strength based on the handgrip or sit-to-stand test criteria, while 50% exhibited low muscle mass, defined either as a reduction in ASMMI below the cut-off values or as a calf circumference below the median. Overall, 2 SpA patients (20%, *p* = 0.47) were classified as sarcopenic versus none among controls.

**Table 1:** Clinical characteristics and therapeutic regimen of the patients included

Patient code	Age at last FU, sex	Disease characteristics	Therapy at baseline	Secondary inefficacy to bDMARDs	Therapy at 36 months	Treatment change	Regular Vitamin D intake
001	55, F	Ax-SpA HLAB27+	adalimumab 40 mg/2 weeks	Yes	ixekizumab, on-demand NSAIDs	Switched from adalimumab to ixekizumab 80 mg/4 weeks	Yes
002	40, M	Ax-SpA HLAB27-	secukinumab 300 mg/4 weeks	No	secukinumab, on-demand NSAIDs	No change	Yes
003	41, M	Ax-SpA HLAB27- with PsO	infliximab ev (prev. adalimumab And secukinumab) 5 mg/kg/8 weeks	Yes	unknown	unknown	No
004	70, M	Ax-SpA HLAB27+	adalimumab 40 mg/2 weeks	No	adalimumab	No change	No
005	52, M	Ax-SpA HLAB27 -	golimumab 50 mg/4 weeks (prev. etanercept, secukinumab)	No	secukinumab	Switched from golimumab to secukinumab 300 mg/4 weeks	No
006	33, M	Ax-SpA HLAB27+	adalimumab 40 mg/2 weeks	Yes	secukinumab	Switched from adalimumab to secukinumab 150 mg/4 weeks	Yes
007	33, M	Ax-SpA with Pso	adalimumab 40 mg/2 weeks + methotrexate 10 mg/week	No	adalimumab	No chang	Yes
008	56, M	Ax-SpA HLAB27+	adalimumab 40 mg/2 weeks	No	adalimumab	No chang	No
009	39, M	Ax-SpA HLAB27 -	infliximab ev 5 mg/kg/8 weeks (prev. adalimumab, certolizumab)	Yes	upadacitinib	Switched from infliximab to upadacitinib 15 mg/die	Yes
010	46, M	Ax-SpA HLAB27+	etanercept 50 mg/week	Yes	None (prev. secukinumab)	Stopped bDMARD	No

**Table 2:** Sample characteristics: comparison between spa patients and controls

Variable	SpA patients (*n* = 10)	Controls (*n* = 10)	*p*-value
Age [years]	43.8 (11.5)	44.9 (16.5)	0.86
Sex, females	1 (10%)	1 (10%)	0.94
Active smoker	1 (10%)	0	0.28
BMI [Kg/m^2^]	25.70 (4.64)	26.13 (2.45)	0.85
*Strength measurements*			
Handgrip test [Kg]	40.67 (15.59)	41.95 (9.32)	0.82
*Body composition*			
% fat	21.26 (4.16)	23.82 (8.15)	0.40
FMI [Kg/m^2^]	5.74 (1.97)	6.35 (2.62)	0.58
ASMMI [Kg/m^2^]	8.93 (1.64)	8.75 (1.03)	0.25
Calf crf [cm]	38.70 (3.65)	37.90 (1.39)	0.53
Arm crf [cm]	29.90 (4.17)	30.30 (2.49)	0.78
Low muscle strength	6 (60%)	1 (10%)	0.30
Low muscle mass	5 (50%)	1 (10%)	0.32
Sarcopenia	2 (20%)	0	0.47
*Densitometric values*			
BMD lumbar [g/cm^2^]	1.11 (0.18)	1.07 (0.13)	0.58
BMD total hip [g/cm^2^]	1.07 (0.15)	0.95 (0.17)	0.09
BMD femur neck [g/cm^2^]	0.89 (0.13)	0.82 (0.12)	0.16
*Calcium-phosphorus metabolism*			
Calcium [mmol/L]	2.41 (0.09)	2.37 (0.07)	0.49
Phosphorus [mmol/L]	0.91 (0.10)	1.11 (0.21)	0.09
PTH [ng/L]	55.00 (43.95;61.65)	26.00 (21.10;62.50)	0.15
Vitamin D [nmol/L]	48.80 (42.60;91.00)	51.00 (27.00;63.00)	0.44

Data are expressed as mean (SD), median (IQR) or numbers (%), as appropriate

*Abbreviations*: *BMI* Body Mass Index, *crf* circumference, *BMD* Bone Mineral Density, *FMI* Fat Mass Index, *ASMMI* Appendicular Skeletal Muscle Mass Index, *PTH *Parathyroid hormone

Table 3 presents the baseline correlation analysis between functional impairment (BASFI, BASDAI, and HAQ scores) and anthropometric, physical performance, and bone-related parameters. BASFI showed strong positive correlations with BMI (*r* = 0.800, *p* < 0.01), fat percentage (*r* = 0.808, *p* < 0.01), and FMI (*r* = 0.903, *p* < 0.01), indicating that higher fat mass and BMI are associated with greater disability. Additionally, we observed moderate — albeit not statistically significant — correlations between sit-to-stand performance (*r* = 0.539) and femoral neck BMD (*r* = 0.537). BASDAI, reflecting disease activity, showed a significant positive correlation with sit-to-stand performance (*r* = 0.677, *p* < 0.05) and fat percentage (*r* = 0.700, *p* < 0.05), suggesting that both poorer lower limb function and higher adiposity may be linked to increased disease activity. However, no significant associations were found between BASDAI and BMD or muscle-related parameters. The HAQ score, which measures overall disability, exhibited strong correlations with calf circumference (*r* = 0.787, *p* < 0.01) and ASMMI (*r* = 0.777, *p* < 0.01). Additionally, HAQ correlated significantly with the 4-meter walking test (*r* = 0.687, *p* < 0.05), reinforcing the relationship between mobility impairment and disability. Correlations with BMI (*r* = 0.407) and fat mass (*r* = 0.581) were moderate but did not reach statistical significance. Analysing the sub-items of the questionnaires (see Supplementary Table 1), we found that total fat percentage and FMI were positively correlated with most items of the BASFI questionnaire. Regarding the HAQ, the items “walking,” “toilet use,” “grip and hand use,” and “daily activities” were positively associated with calf circumference And the 4-meter walking test score, indicating that a larger calf circumference And better performance in the 4-meter walking test were linked to greater functional ability in these daily activities.

**Table 3:** Simple correlations between spa disease questionnaire and bone and body composition parameters

	BMI	Calf crf	Arm crf	4 m walking test	Sit-to-stand	handgrip	BMD LUMBAR	BMD HIP	BMD FEMUR NECK	% FAT	FMI	ASMMI
*BASFI total*	*0.800* ^**^	0.596	0.489	0.418	0.539	−0.193	0.194	0.382	0.537	*0.808* ^**^	*0.903* ^**^	0.311
*BASDAI total*	0.284	0.263	−0.048	0.112	*0.677*	−0.297	−0.002	0.207	0.352	*0.700*	0.542	−0.097
*HAQ total*	0.407	*0.787* ^**^	0.598	*0.687* ^*^	0.535	−0.480	0.376	0.378	0.481	0.581	0.531	*0.777* ^**^

*Abbreviations*: *BMI* Body Mass Index, *crf* circumference, *4m walking test* 4-Meter Walking Test, *BMD* Bone Mineral Density, *FMI* Fat Mass Index, *ASMMI* Appendicular Skeletal Muscle Mass Index, *BASFI* Bath Ankylosing Spondylitis Functional Index, *BASDAI* Bath Ankylosing Spondylitis Disease Activity Index, *HAQ* Health Assessment Questionnaire

*Correlation is significant at the 0.05 level (2-tailed)

**Correlation is significant at the 0.01 level (2-tailed)

**Fig. 1:**
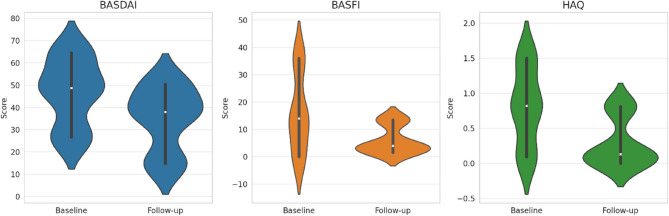
Distribution of scores between baseline and follow-up for each questionnaire. *Abbreviations*: BASFI = Bath Ankylosing Spondylitis Functional Index; BASDAI = Bath Ankylosing Spondylitis Disease Activity Index; HAQ = Health Assessment Questionnaire

Patients were re-evaluated at follow-up after a mean period of 36 months. At that time, the treatment outcomes were as follows: 4 patients (40%) remained on the same bDMARD, 4 patients (40%) switched to a different class of bDMARDs, 1 patient (10%) discontinued treatment due to remission, And for 1 patient (10%), data on the last treatment regimen were unavailable. At follow-up assessment, no significant differences were observed in body composition, strength measurements, or bone parameters, except for an improvement in sit-to-stand scores [from 17.37 (7.47) to 11.98 (3.81), *p* = 0.02] and a slight increase in femur neck BMD values [from 0.89 (0.13) to 1.02 (0.14), *p* = 0.01] (Table 4). Moreover, the percentage of patients with reduced muscle strength And low muscle mass had decreased to 33.3% (*p* = 0.17) And 44.4% (*p* = 0.52), respectively. Regarding disease activity questionnaires, the only significant difference observed was in the BASDAI score [from 48.75 (26.63;64.50) to 38.00 (14.75;50.50), *p* = 0.04], while no significant differences were found for BASFI [from 14.0 (0;36.0) to 4.0 (1.5;13.5), *p* = 0.80] and HAQ [from 0.82 (0.09;1.50) to 0.13 (0;0.81), *p* = 0.13] (Fig. 1). Moreover, no significant differences in calcium-phosphorus metabolism were observed and we recorded no vertebral fractures during the follow-up (data not shown).

**Table 4:** Body compositions, bone parameters and strength measurements changes from baseline to follow-up

Variable	Baseline	Follow-up	*p*-value
BMI [kg/m^2^]	25.70 (4.64)	25.48 (4.04)	0.37
*Strength measurements*			
Handgrip test [Kg]	40.67 (15.59)	44.92 (11.54)	0.26
Sit-to-stand test [sec]	17.37 (7.47)	11.98 (3.81)	*0.02*
*Body composition*			
%fat	21.26 (4.16)	22.30 (5.79)	0.14
FMI [kg/m^2^]	5.74 (1.97)	5.61 (2.80)	0.86
ASMMI [kg/m^2^]	8.93 (1.64)	9.05 (1.37)	0.48
Calf crf [cm]	38.70 (3.65)	37.11 (3.26)	0.43
Arm crf [cm]	29.90 (4.17)	29.67 (3.71)	0.89
*Densitometric values*			
BMD lumbar [g/cm^2^]	1.11 (0.18)	1.17 (0.21)	0.13
BMD total hip [g/cm^2^]	1.07 (0.15)	0.96 (0.14)	*0.007*
BMD femur neck [g/cm^2^]	0.89 (0.13)	1.02 (0.14)	*0.01*

Numbers are expressed as mean (SD)

*Abbreviations*: *BMI* Body Mass Index, *crf* circumference, *BMD* Bone Mineral Density, *FMI* Fat Mass Index, *ASMMI* Appendicular Skeletal Muscle Mass Index

**Fig. 2:**
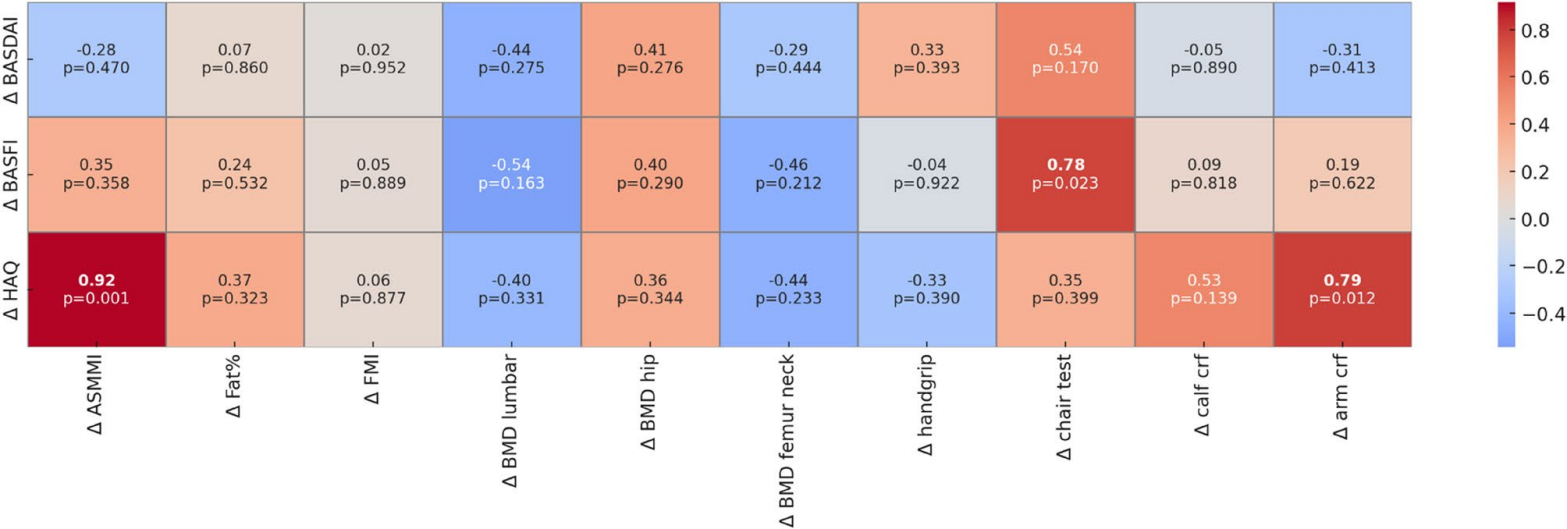
Simple correlation between variation of SpA disease questionnaire and variations in bone and body composition parameters. *Abbreviations*: BMI = Body Mass Index; BMD = Bone Mineral Density; FMI = Fat Mass Index; ASMMI = Appendicular Skeletal Muscle Mass Index; BASFI = Bath Ankylosing Spondylitis Functional Index; BASDAI = Bath Ankylosing Spondylitis Disease Activity Index; HAQ = Health Assessment Questionnaire

There was a significant positive correlation between variation in sit-to-stand questionnaire and variations in BASFI scores (*r* = 0.78, *p* < 0.01), as well as between variations in ASMMI and variations in HAQ scores (*r* = 0.92, *p* < 0.001) (Fig. 2). The analysis of the correlations between changes in functional ability scores (sub-items of BASFI, BASDAI, and HAQ) And variations in bone And body composition parameters are reported in Supplementary Table 2. For BASFI, improvements in the ability to reach a shelf at shoulder height were strongly correlated with increased ASMMI (*r* = 0.90, *p* < 0.001) and arm circumference (*r* = 0.96, *p* < 0.001). Additionally, the ability to stand up from the floor without assistance was significantly associated with improvements in sit-to-stand performance (*r* = 0.93, *p* < 0.001). Regarding BASDAI, greater reductions in pain levels, particularly in the neck, back, and hips, were associated with improvements in lumbar BMD (*r*=−0.735, *p* = 0.04). Additionally, reductions in morning stiffness duration and intensity showed significant positive correlations with improved sit-to-stand performance (*r* = 0.798, *p* = 0.02), suggesting a potential link between disease activity and functional mobility. For HAQ, better dressing ability and grip and hand use were significantly associated with higher ASMMI and arm circumference. Similarly, improved toilet use and personal hygiene scores correlated with greater arm circumference. Walking ability was negatively linked to higher BMD in the lumbar spine (*r*=−0.718, *p* = 0.045) and femur neck (*r*=−0.707, *p* = 0.033). Finally, the ability to perform daily activities was significantly associated with greater ASMMI (*r* = 0.712, *p* = 0.03).

## Discussion

Our study, the first with such a long follow-up period, analysed the correlation between body composition and questionnaire scores related to disease activity and quality of life (QoL) in patients with SpA. Our data show that a higher fat mass is associated with increased disease activity. The BASDAI is a patient-reported index that assesses fatigue, spinal and peripheral joint pain, localized tenderness, and morning stiffness. While it reflects subjective disease burden, it does not directly measure systemic inflammation (e.g., through CRP). Therefore, changes in BASDAI scores may reflect not only biological activity but also perceived pain, stiffness, and disability. These components can influence physical function and interact with body composition parameters, making the interpretation of correlations complex. While reductions in disease activity were generally accompanied by improvements in functional performance (e.g., sit-to-stand test), the relationship with muscle mass parameters appeared less consistent. In particular, we observed a counterintuitive positive correlation between ASMMI and HAQ, suggesting that greater muscle mass was associated with increased perceived disability in this cohort. This paradox may be explained by qualitative alterations in muscle tissue—such as fat infiltration, fibrosis, or inflammation—that are not captured by volumetric measurements alone. Moreover, pain, joint stiffness, and neuromuscular dysfunction may impair function independently of muscle quantity, particularly in chronic inflammatory conditions like ax-SpA.

Only a few studies have examined the impact of body composition on function or disease activity in SpA patients [16–18]. Recently, there has been growing interest in conditions such as osteoporosis and sarcopenia among these patients, since factors like age, male sex, degree of inflammation, BMI, lack of physical exercise, and the use of certain medications can negatively affect both muscle mass and bone density [19]. Although increasing evidence indicates that muscle strength and mass often decline in patients with axial SpA, the prevalence of sarcopenia—a condition marked by impaired muscle health—has not yet been fully defined in this population. A recent systematic review [10] carried out on ten studies highlighted that muscle strength is generally reduced in patients with RD, including those with SpA. Although strength measurements were performed using various tools (e.g., handgrip dynamometer, knee-extension device, isokinetic dynamometer), the overall trend indicated a decline in muscle strength among SpA patients. Regarding sarcopenia, data are more contrasting. A large systematic review with meta-analysis [20] encompassing 16 studies And 999 SpA patients revealed An overall sarcopenia prevalence of 25.0% (95% confidence interval: 0.127 to 0.352). Other studies found slightly lower rates depending on criteria or population, such as 22.1% [21] or as low as 5.1% in PsA [22]. In our cohort, the prevalence of sarcopenia was approximately 20.0%. However, changes in body composition occur even before sarcopenia is fully established. These early alterations are likely influenced by the same factors that contribute to SpA pathology, including the underlying inflammatory state. Longitudinal studies have shown that high values of fat mass, BMI, or FMI are associated with increased inflammatory markers over time. Obesity, itself a low-grade inflammatory condition, is characterised by infiltration of immune cells into adipose tissue, where adipocytes and white blood cells release pro-inflammatory cytokines. This pro-inflammatory milieu may contribute to altered muscle and bone metabolism [23]. These findings underscore the importance of studying the relationship between body composition parameters and disease activity in SpA. Alterations in body composition—leading to an imbalance between lean and fat mass—may compromise not only physical function and QoL, but also perpetuate inflammation and further impair performance.

In line with this hypothesis, our study demonstrated that higher BASDAI or BASFI scores were associated with higher BMI, fat mass, or FMI, as well as worse performance on the sit-to-stand test. These findings align with prior studies [17, 18], although discrepancies exist [16]. In contrast to earlier research, our study also evaluated physical performance directly. At baseline, lower gait speed was associated with higher HAQ scores, reinforcing the link between physical function and QoL [24].

A particularly unexpected finding was the positive correlation between HAQ scores and muscle mass indicators (ASMMI and calf circumference). While one would expect that higher muscle mass corresponds with better function, this was not the case. A plausible explanation lies in the distinction between muscle quantity and quality. In chronic inflammatory settings, muscle tissue may appear preserved in volume but compromised in quality due to fat infiltration (myosteatosis), reduced capillary density, or inflammatory myopathy. This phenomenon has been observed in rheumatoid arthritis and IBD. Moreover, joint pain and stiffness may contribute to disability independently of muscle mass. Future studies incorporating imaging (e.g., muscle MRI or ultrasound), electromyography, and inflammatory profiling are needed to clarify the interplay between muscle quantity, quality, and function in SpA., Importantly, it is possible that patients with preserved or even elevated muscle mass still experience functional limitations due to pain or neuromuscular alterations that are not captured by DXA-derived indices.

At 36-months, we observed significant improvements in physical performance and functional capacity. Muscle strength and mass correlated with bettersit-to-stand performance, in parallel with lower BASDAI and BASFI scores. In other words, lower disease activity appears to be closely linked with enhanced physical function. The marked improvements observed in the sit-to-stand test underscore the importance of lower-limb strength and mobility. Specifically, better performance on this test was associated with an increased ability to stand up from the floor and a reduction in morning stiffness, further supporting the benefits of enhanced lower-body strength. In addition, an increase in ASMMI correlated with lower HAQ scores, indicating a possible protective role of muscle mass over time. More detailed analyses revealed that tasks such as reaching a shelf at shoulder height, dressing, and grip strength were positively associated with both ASMMI and arm circumference—highlighting the critical role of upper-limb muscle mass in maintaining independence. Furthermore, the positive relationship between arm circumference and the ability to perform self-care activities, such as using the toilet and maintaining hygiene, reinforces the importance of upper-body strength in daily living. An unexpected finding was that better walking performance correlated with lower BMD at the lumbar spine and femur neck. This could reflect measurement limitations of DXA in patients with new bone formation or postural abnormalities. Alternatively, increased mobility may not always protect against localized bone loss, depending on mechanical loading patterns.

However, our results should also be interpreted in light of the pharmacological treatment. Most of the patients in this study were treated with tumour necrosis factor (TNF)-alpha inhibitors and interleukin (IL)−17 inhibitors. These treatments appear to slow bone and muscle mass loss while preserving strength, as previously reported in the literature [25]. In terms of bone health, the use of bDMARDs has been shown to modify and slow the progression of bone loss. It is well-documented that anti-TNF-alpha agents can reduce bone loss, contributing not only to overall clinical improvements in ax-SpA but also to increased BMD [25]. For instance, significant increases in BMD at the spine and hip following treatment with infliximab were reported several years ago in ax-SpA patients (ASSERT trial) [26]. Although only a few studies have assessed the long-term effects of TNF-alpha inhibitors, evidence suggests that treatment with these drugs can improve BMD, although the risk of fractures remains unchanged [27, 28]. A study by Beauger et al. [29] reported increased trabecular BMD in 46 patients with ax-SpA, albeit with no change in cortical BMD over five years. This finding may be explained by the differential effect of TNF-alpha inhibitors on trabecular versus cortical bone. Similarly, in our cohort, BMD at the femoral neck, which reflects trabecular bone, improved after 36 months, while BMD at the total hip showed a trend toward worsening, likely reflecting a reduced effect of TNF-alpha inhibitors on cortical bone. Although BMD at the lumbar spine remained stable over 36 months of follow-up, we observed a positive correlation between the pain items of the BASDAI, and an amelioration in lumbar BMD, suggesting that pain reduction, which may have led to an increase in physical exercise, could potentially have contributed to the observed improvement of the lumbar BMD. The IL-17 A inhibitor secukinumab also appears to preserve BMD in patients with SpA [30, 31]. Recent studies have shown minimal radiographic spinal progression after 2 years of follow-up in patients treated with secukinumab [32], confirming earlier clinical findings [33, 34]. However, secukinumab appears to have only a marginal effect on bone turnover markers. It is worth noting that measurement of lumbar spine BMD using DXA in SpA patients may be controversial due to false elevation caused by bone proliferation or new bone formation, a common feature in SpA [35]. In vitro studies have also demonstrated that inhibiting IL-17 A suppresses osteogenic differentiation and significantly reduces mineralization. In our cohort, treatment with secukinumab was overall associated with stability in BMD. Notably, all our patients were treated with bDMARDs. The effects of anti-TNF-alpha treatment on muscle strength have been well-documented, with significant improvements observed in functional limitations, performance, and muscle endurance over time [36]. It is noteworthy that none of our patients were receiving glucocorticoids, which are known to negatively affect both bone and muscle metabolism. A recent systematic review investigating the impact of bDMARDs on muscle mass in rheumatoid arthritis (RA) and SpA also confirmed improvements in muscle strength. However, the overall effect on total lean mass was not significant in either RA or SpA. We could not assess the impact of physical exercise in our cohort, which likely influenced in a consistent way muscle performance. Indeed, as per recent EULAR recommendations [37], physical exercise should be an integral part of the treatment plan due to its beneficial effects in reducing pro-inflammatory cytokines, and ameliorating the physical performance, in turn reflecting an improvement on muscle strength. Thus, a comprehensive approach that combines pharmacologic treatment and physical exercise may result in better outcomes in SpA patients.

The main limitation of this study is the small sample size. No formal sample size calculation was performed prior to recruitment, given the exploratory nature of this pilot study. As a result, the statistical power may be limited, And findings should be interpreted with caution. Nonetheless, the study provides useful preliminary data to inform future, adequately powered trials. However, to the best of our knowledge, this is one of the first studies to assess the follow-up of muscle And bone parameters in SpA patients treated with bDMARDs over a 36-month period. Additionally, there may be biases related to the composition of the sample, as 90% of the participants were males, which could have potentially led to an overestimation of muscle strength and an underestimation of bone loss. This underrepresentation of women is a notable limitation, especially given known sex differences in muscle mass, hormonal influences, and the clinical manifestation of sarcopenia. The skewed sample may have affected the observed prevalence and characterization of sarcopenia, possibly underestimating its true burden in female patients with SpA. Future studies should aim for a more balanced gender distribution and consider conducting sex-stratified analyses to better capture the differential impact of SpA on musculoskeletal outcomes in men and women. Moreover, while improvements in muscle strength and physical performance were observed, we did not collect specific data on physical activity or rehabilitation during the study. As such, any inference about increased physical activity contributing to the observed improvements remains speculative. Future research should include structured and objective assessments of physical activity—such as exercise questionnaires, logs, or wearable devices—to better clarify its role in disease outcomes. Another limitation concerns the assessment of disease activity. Although the BASDAI is a commonly used patient-reported outcome, it is subjective and can be affected by non-inflammatory symptoms. No inflammatory biomarkers such as CRP or ESR were collected at both timepoints, and ASDAS-CRP was not employed. This limits the objectivity and granularity in evaluating the inflammatory burden and its association with body composition. Future studies should incorporate composite indices like ASDAS-CRP to strengthen the correlation between disease activity and changes in muscle or bone parameters. Finally, we emphasize that all associations reported are correlational in nature. Due to the observational design and small sample, no causal inferences can be drawn. Larger, prospective studies are needed to elucidate mechanisms linking inflammation, body composition, and function in ax-SpA. Nonetheless, this study may serve as a cornerstone toward the development of a more comprehensive diagnostic framework, one that should incorporate the evaluation of both muscle and bone metabolism from the onset. Such an approach would be invaluable for optimising clinical management. Furthermore, the prescription of physical exercise should be considered a fundamental aspect of treatment, given its potential to enhance inflammatory control and overall patient reported outcomes (PROs) for both pain and physical performance. 

In conclusion, our study suggests that improved body composition, particularly lower fat mass and preserved or increased muscle mass, is associated with reduced disease activity and better functional performance in ax-SpA. These results support a multimodal treatment approach that integrates pharmacologic therapy with physical activity interventions aimed at preserving musculoskeletal health and quality of life.

## Supplementary Information

Supplementary Material 1.

## Data Availability

The datasets generated and analyzed during the current study are not publicly available due to privacy reason but are available from the corresponding author on reason request.
