# Supplementary material for: Body composition, bone mineral density, and functional impairment in axial spondyloarthritis: a 36-month longitudinal study
Source: BMC Musculoskelet Disord. 2025 Sep 30;26:878. doi: 10.1186/s12891-025-09088-8 (PMC12486735; doi:10.1186/s12891-025-09088-8)
Supplement: Supplementary file 1 — Supplementary Material 1. [file 12891_2025_9088_MOESM1_ESM.docx]

**Supplementary Table S1.** **Simple correlations between SpA disease questionnaire sub-items and bone and body composition parameters.**

|  |  | | *BMI* | *Calf crf* | *Arm crf* | *4m walking test* | *Sit-to-stand* | *handgrip* | *BMD LUMBAR* | *BMD HIP* | *BMD FEMUR NECK* | *% FAT* | *FMI* | *ASMMI* |
| --- | --- | --- | --- | --- | --- | --- | --- | --- | --- | --- | --- | --- | --- | --- |
| BASFI (Bath Ankylosing Spondylitis Functional Index) | Putting on socks or stockings without help | Pearson Correlation | 0.405 | -0.023 | -0.094 | -0.089 | 0.284 | -0.179 | -0.328 | -0.043 | 0.221 | 0.731^*^ | 0.692^*^ | -0.176 |
|  |  | p-value | 0.246 | 0.950 | 0.796 | 0.820 | 0.427 | 0.620 | 0.355 | 0.906 | 0.540 | 0.016 | 0.027 | 0.627 |
|  | Bending forward from the waist to pick up an object from the floor | Pearson Correlation | 0.558 | 0.548 | 0.443 | 0.440 | 0.223 | -0.312 | -0.032 | 0.134 | 0.310 | 0.641^*^ | 0.707^*^ | 0.485 |
|  |  | p-value | 0.094 | 0.101 | 0.200 | 0.236 | 0.536 | 0.380 | 0.931 | 0.711 | 0.384 | 0.046 | 0.022 | 0.156 |
|  | Reaching a shelf at shoulder height without help | Pearson Correlation | 0.594 | 0.525 | 0.432 | 0.272 | 0.054 | -0.148 | 0.001 | 0.234 | 0.381 | 0.568 | 0.708^*^ | 0.415 |
|  |  | p-value | 0.070 | 0.119 | 0.213 | 0.479 | 0.882 | 0.684 | 0.999 | 0.516 | 0.278 | 0.087 | 0.022 | 0.233 |
|  | Getting up from a chair without using hands for support | Pearson Correlation | 0.788^**^ | 0.439 | 0.341 | 0.201 | 0.294 | 0.127 | 0.042 | 0.244 | 0.316 | 0.501 | 0.736^*^ | 0.000 |
|  |  | p-value | 0.007 | 0.204 | 0.336 | 0.604 | 0.409 | 0.726 | 0.909 | 0.496 | 0.374 | 0.140 | 0.015 | 0.999 |
|  | Standing up from the floor without assistance | Pearson Correlation | 0.465 | 0.176 | 0.118 | 0.292 | 0.822^**^ | -0.235 | 0.080 | 0.176 | 0.396 | 0.795^**^ | 0.671^*^ | -0.013 |
|  |  | p-value | 0.176 | 0.627 | 0.744 | 0.446 | 0.004 | 0.514 | 0.826 | 0.627 | 0.257 | 0.006 | 0.034 | 0.972 |
|  | Climbing 12 steps without using a handrail | Pearson Correlation | 0.730^*^ | 0.313 | 0.249 | 0.134 | 0.154 | 0.086 | -0.184 | 0.077 | 0.233 | 0.560 | 0.769^**^ | -0.012 |
|  |  | p-value | 0.016 | 0.378 | 0.488 | 0.730 | 0.672 | 0.813 | 0.610 | 0.833 | 0.517 | 0.092 | 0.009 | 0.973 |
|  | Looking over the shoulder without turning the whole body | Pearson Correlation | 0.824^**^ | 0.687^*^ | 0.704^*^ | 0.569 | 0.403 | 0.015 | 0.577 | 0.660^*^ | 0.654^*^ | 0.535 | 0.731^*^ | 0.428 |
|  |  | p-value | 0.003 | 0.028 | 0.023 | 0.110 | 0.248 | 0.967 | 0.080 | 0.038 | 0.040 | 0.111 | 0.016 | 0.217 |
|  | Walking long distances without interruptions (variable distance) | Pearson Correlation | 0.509 | 0.568 | 0.395 | 0.141 | 0.172 | -0.134 | 0.432 | 0.563 | 0.472 | 0.333 | 0.477 | 0.241 |
|  |  | p-value | 0.133 | 0.087 | 0.258 | 0.717 | 0.635 | 0.713 | 0.212 | 0.090 | 0.169 | 0.348 | 0.163 | 0.503 |
|  | Performing daily activities such as dressing or turning in bed | Pearson Correlation | 0.579 | 0.695^*^ | 0.591 | 0.726^*^ | 0.773^**^ | -0.305 | 0.391 | 0.403 | 0.554 | 0.714^*^ | 0.676^*^ | 0.586 |
|  |  | p-value | 0.079 | 0.026 | 0.072 | 0.027 | 0.009 | 0.391 | 0.264 | 0.249 | 0.097 | 0.020 | 0.032 | 0.075 |
|  | Overall perception of the ability to manage daily activities | Pearson Correlation | .759^*^ | 0.462 | 0.418 | 0.493 | 0.708^*^ | -0.339 | 0.101 | 0.220 | 0.417 | 0.906^**^ | 0.912^**^ | 0.247 |
|  |  | p-value | 0.011 | 0.179 | 0.230 | 0.178 | 0.022 | 0.339 | 0.782 | 0.542 | 0.230 | 0.000 | 0.000 | 0.492 |
| BASDAI (Bath Ankylosing Spondylitis Disease Activity Index) | Fatigue | Pearson Correlation | -0.048 | 0.083 | -0.347 | -0.035 | 0.604 | -0.434 | -0.171 | -0.047 | 0.072 | 0.490 | 0.236 | -0.237 |
|  |  | p-value | 0.894 | 0.820 | 0.326 | 0.930 | 0.065 | 0.210 | 0.638 | 0.898 | 0.843 | 0.150 | 0.511 | 0.509 |
|  | Pain in the neck, back, and hips | Pearson Correlation | 0.379 | 0.430 | 0.059 | 0.112 | 0.540 | -0.381 | 0.026 | 0.262 | 0.349 | 0.674^*^ | 0.603 | 0.009 |
|  |  | p-value | 0.280 | 0.215 | 0.872 | 0.773 | 0.107 | 0.277 | 0.943 | 0.464 | 0.323 | 0.033 | 0.065 | 0.979 |
|  | Pain or swelling in other joints | Pearson Correlation | 0.479 | 0.139 | 0.319 | 0.376 | 0.763^*^ | -0.136 | 0.304 | 0.289 | 0.441 | 0.627 | 0.571 | 0.109 |
|  |  | p-value | 0.161 | 0.702 | 0.369 | 0.319 | 0.010 | 0.709 | 0.394 | 0.418 | 0.202 | 0.052 | 0.084 | 0.763 |
|  | Feeling of discomfort or tenderness in sensitive areas | Pearson Correlation | -0.290 | -0.202 | -0.595 | -0.361 | 0.099 | -0.401 | -0.686^*^ | -0.388 | -0.229 | 0.331 | 0.105 | -0.413 |
|  |  | p-value | 0.417 | 0.576 | 0.069 | 0.341 | 0.785 | 0.251 | 0.029 | 0.267 | 0.524 | 0.350 | 0.773 | 0.236 |
|  | Duration of morning stiffness | Pearson Correlation | 0.293 | 0.207 | 0.066 | 0.222 | 0.746^*^ | -0.159 | 0.270 | 0.330 | 0.425 | 0.575 | 0.430 | -0.046 |
|  |  | p-value | 0.412 | 0.566 | 0.856 | 0.565 | 0.013 | 0.660 | 0.450 | 0.351 | 0.221 | 0.082 | 0.215 | 0.900 |
|  | Intensity of morning stiffness | Pearson Correlation | 0.504 | 0.528 | 0.450 | 0.364 | 0.307 | 0.405 | 0.547 | 0.643^*^ | 0.625 | 0.287 | 0.375 | 0.241 |
|  |  | p-value | 0.138 | 0.116 | 0.192 | 0.335 | 0.388 | 0.246 | 0.102 | 0.045 | 0.053 | 0.422 | 0.286 | 0.502 |
| HAQ (Health Assessment Questionnaire) | Dressing | Pearson Correlation | 0.423 | 0.570 | 0.492 | 0.399 | 0.339 | -0.374 | 0.317 | 0.434 | 0.570 | 0.650^*^ | 0.612 | 0.612 |
|  |  | p-value | 0.223 | 0.085 | 0.148 | 0.287 | 0.339 | 0.286 | 0.373 | 0.210 | 0.085 | 0.042 | 0.060 | 0.060 |
|  | Eating | Pearson Correlation | 0.264 | 0.552 | 0.192 | 0.178 | 0.450 | -0.391 | 0.284 | 0.429 | 0.455 | 0.488 | 0.431 | 0.262 |
|  |  | p-value | 0.461 | 0.098 | 0.595 | 0.647 | 0.191 | 0.264 | 0.427 | 0.216 | 0.187 | 0.152 | 0.214 | 0.465 |
|  | Personal hygiene | Pearson Correlation | 0.456 | 0.628 | 0.494 | 0.527 | 0.300 | -0.320 | 0.112 | 0.237 | 0.408 | 0.597 | 0.612 | 0.623 |
|  |  | p-value | 0.185 | 0.052 | 0.147 | 0.145 | 0.400 | 0.368 | 0.758 | 0.510 | 0.242 | 0.068 | 0.060 | 0.054 |
|  | Getting up | Pearson Correlation | 0.008 | 0.571 | 0.432 | 0.771^*^ | 0.562 | -0.423 | 0.309 | 0.152 | 0.246 | 0.281 | 0.105 | 0.743^*^ |
|  |  | p-value | 0.983 | 0.085 | 0.213 | 0.015 | 0.091 | 0.223 | 0.384 | 0.676 | 0.493 | 0.432 | 0.773 | 0.014 |
|  | Walking | Pearson Correlation | 0.651^*^ | 0.717^*^ | 0.682^*^ | 0.527 | 0.401 | -0.249 | 0.535 | 0.639^*^ | 0.709^*^ | 0.661^*^ | 0.725^*^ | 0.642^*^ |
|  |  | p-value | 0.041 | 0.020 | 0.030 | 0.145 | 0.250 | 0.488 | 0.111 | 0.047 | 0.022 | 0.038 | 0.018 | 0.045 |
|  | Toilet use | Pearson Correlation | 0.299 | 0.663^*^ | 0.423 | 0.707^*^ | 0.764^*^ | -0.612 | 0.244 | 0.208 | 0.342 | 0.646^*^ | 0.487 | 0.611 |
|  |  | p-value | 0.402 | 0.037 | 0.224 | 0.033 | 0.010 | 0.060 | 0.497 | 0.564 | 0.333 | 0.044 | 0.154 | 0.061 |
|  | Grip and hand use | Pearson Correlation | 0.117 | 0.729^*^ | 0.617 | 0.675^*^ | 0.160 | -0.336 | 0.400 | 0.312 | 0.325 | 0.160 | 0.137 | .919^**^ |
|  |  | p-value | 0.747 | 0.017 | 0.057 | 0.046 | 0.658 | 0.342 | 0.252 | 0.381 | 0.359 | 0.660 | 0.705 | 0.000 |
|  | Daily activities | Pearson Correlation | 0.593 | 0.746^*^ | 0.579 | 0.781^*^ | 0.599 | -0.489 | 0.244 | 0.107 | 0.174 | 0.501 | 0.561 | 0.614 |
|  |  | p-value | 0.071 | 0.013 | 0.079 | 0.013 | 0.067 | 0.151 | 0.497 | 0.770 | 0.630 | 0.140 | 0.092 | 0.059 |

*Abbreviations*: BMI = Body Mass Index; crf = circumference; 4m walking test = 4-Meter Walking Test; BMD = Bone Mineral Density; FMI = Fat Mass Index; ASMMI = Appendicular Skeletal Muscle Mass Index.

**Supplementary Table S2. Simple correlations between variations in BASFI, BASDAI and HAQ sub-items and variations in bone and body composition parameters.**

|  |  | | *Δ ASMMI* | *Δ fat* | *Δ FMI* | *Δ BMD lumbar* | *Δ BMD hip* | *Δ BMD femur neck* | *Δ handgrip* | *Δ sit-to.stand* | *Δ calf crf* | *Δ arm crf* |
| --- | --- | --- | --- | --- | --- | --- | --- | --- | --- | --- | --- | --- |
| *BASFI* | Δ Putting on socks or stockings without help | Pearson Correlation | -0.149 | -0.040 | 0.056 | 0.199 | -0.073 | 0.057 | 0.163 | 0.581 | -0.138 | -0.090 |
|  |  | p-value | 0.701 | 0.918 | 0.887 | 0.636 | 0.851 | 0.885 | 0.675 | 0.131 | 0.724 | 0.817 |
|  | Δ Bending forward from the waist to pick up an object from the floor | Pearson Correlation | -0.082 | 0.547 | 0.519 | -0.461 | -0.232 | -0.158 | -0.003 | -0.460 | -0.079 | -0.067 |
|  |  | p-value | 0.833 | 0.127 | 0.152 | 0.250 | 0.547 | 0.684 | 0.993 | 0.252 | 0.840 | 0.864 |
|  | Δ Reaching a shelf at shoulder height without help | Pearson Correlation | 0.940^**^ | 0.442 | 0.234 | -0.043 | 0.344 | -0.416 | -0.118 | 0.101 | 0.589 | 0.960^**^ |
|  |  | p-value | 0.000 | 0.234 | 0.545 | 0.919 | 0.365 | 0.265 | 0.762 | 0.812 | 0.095 | 0.000 |
|  | Δ Getting up from a chair without using hands for support | Pearson Correlation | -0.134 | 0.375 | 0.235 | -0.604 | -0.060 | -0.147 | 0.052 | 0.282 | -0.011 | -0.194 |
|  |  | p-value | 0.731 | 0.320 | 0.542 | 0.113 | 0.878 | 0.706 | 0.894 | 0.499 | 0.977 | 0.616 |
|  | Δ Standing up from the floor without assistance | Pearson Correlation | 0.150 | -0.095 | -0.152 | -0.048 | 0.279 | -0.185 | 0.073 | 0.930^**^ | -0.038 | 0.066 |
|  |  | p-value | 0.701 | 0.808 | 0.696 | 0.910 | 0.467 | 0.635 | 0.852 | 0.001 | 0.923 | 0.867 |
|  | Δ Climbing 12 steps without using a handrail | Pearson Correlation | 0.215 | 0.661 | 0.573 | -0.189 | 0.128 | -0.362 | 0.334 | 0.089 | 0.158 | 0.327 |
|  |  | p-value | 0.579 | 0.053 | 0.107 | 0.654 | 0.743 | 0.338 | 0.380 | 0.834 | 0.684 | 0.390 |
|  | Δ Looking over the shoulder without turning the whole body | Pearson Correlation | 0.404 | 0.411 | 0.118 | -0.851^**^ | 0.402 | -0.559 | -0.133 | 0.426 | 0.090 | 0.174 |
|  |  | p-value | 0.281 | 0.272 | 0.762 | 0.007 | 0.284 | 0.118 | 0.733 | 0.292 | 0.817 | 0.655 |
|  | Δ Walking long distances without interruptions (variable distance) | Pearson Correlation | 0.008 | 0.059 | -0.107 | -0.732^*^ | 0.495 | -0.448 | -0.048 | 0.584 | -0.088 | -0.210 |
|  |  | p-value | 0.983 | 0.880 | 0.785 | 0.039 | 0.175 | 0.227 | 0.903 | 0.129 | 0.822 | 0.588 |
|  | Δ Performing daily activities such as dressing or turning in bed | Pearson Correlation | 0.732^*^ | 0.255 | 0.096 | -0.393 | 0.377 | -0.478 | -0.183 | 0.630 | 0.317 | 0.605 |
|  |  | p-value | 0.025 | 0.507 | 0.806 | 0.335 | 0.318 | 0.193 | 0.637 | 0.094 | 0.405 | 0.084 |
|  | Δ Overall perception of the ability to manage daily activities | Pearson Correlation | 0.335 | -0.027 | -0.262 | -0.476 | 0.595 | -0.442 | 0.014 | 0.842^**^ | 0.040 | 0.110 |
|  |  | p-value | 0.378 | 0.946 | 0.497 | 0.233 | 0.091 | 0.234 | 0.972 | 0.009 | 0.918 | 0.778 |
| *BASDAI* | Δ Fatigue | Pearson Correlation | -0.372 | 0.581 | 0.304 | -0.493 | -0.039 | -0.061 | 0.583 | -0.039 | 0.031 | -0.323 |
|  |  | p-value | 0.324 | 0.101 | 0.427 | 0.214 | 0.920 | 0.877 | 0.099 | 0.927 | 0.936 | 0.397 |
|  | Δ Pain in the neck, back, and hips | Pearson Correlation | -0.131 | 0.103 | -0.165 | -.735^*^ | 0.229 | -0.126 | 0.073 | 0.233 | 0.194 | -0.265 |
|  |  | p-value | 0.738 | 0.793 | 0.672 | 0.038 | 0.553 | 0.746 | 0.853 | 0.579 | 0.617 | 0.491 |
|  | Δ Pain or swelling in other joints | Pearson Correlation | -0.139 | -0.165 | 0.131 | -0.140 | 0.736^*^ | -0.666 | 0.210 | 0.676 | -0.590 | -0.212 |
|  |  | p-value | 0.721 | 0.672 | 0.737 | 0.741 | 0.024 | 0.050 | 0.587 | 0.065 | 0.094 | 0.584 |
|  | Δ Feeling of discomfort or tenderness in sensitive areas | Pearson Correlation | -0.259 | -0.356 | -0.072 | 0.690 | -0.016 | 0.282 | 0.177 | 0.197 | 0.302 | 0.001 |
|  |  | p-value | 0.500 | 0.347 | 0.855 | 0.058 | 0.967 | 0.463 | 0.649 | 0.641 | 0.430 | 0.999 |
|  | Δ Duration of morning stiffness | Pearson Correlation | -0.035 | 0.065 | -0.034 | -0.429 | 0.456 | -0.395 | 0.187 | 0.798^*^ | -0.154 | -0.165 |
|  |  | p-value | 0.929 | 0.867 | 0.931 | 0.288 | 0.217 | 0.292 | 0.630 | 0.018 | 0.692 | 0.672 |
|  | Δ Intensity of morning stiffness | Pearson Correlation | -0.045 | 0.182 | -0.075 | -0.824^*^ | 0.330 | -0.363 | 0.006 | 0.486 | -0.117 | -0.285 |
|  |  | p-value | 0.909 | 0.638 | 0.847 | 0.012 | 0.386 | 0.338 | 0.987 | 0.222 | 0.764 | 0.457 |
| *HAQ* | Δ Dressing | Pearson Correlation | 0.920^**^ | 0.332 | 0.045 | -0.448 | 0.403 | -0.494 | -0.355 | 0.324 | 0.457 | 0.759^*^ |
|  |  | p-value | 0.000 | 0.383 | 0.908 | 0.266 | 0.282 | 0.176 | 0.349 | 0.434 | 0.216 | 0.018 |
|  | Δ Eating | Pearson Correlation | 0.419 | 0.226 | -0.144 | -0.609 | 0.247 | -0.198 | -0.137 | 0.559 | 0.519 | 0.284 |
|  |  | p-value | 0.262 | 0.558 | 0.712 | 0.109 | 0.522 | 0.610 | 0.726 | 0.150 | 0.152 | 0.458 |
|  | Δ Personal hygiene | Pearson Correlation | 0.743^*^ | -0.051 | -0.176 | 0.293 | 0.395 | -0.191 | -0.087 | 0.306 | 0.476 | 0.751^*^ |
|  |  | p-value | 0.022 | 0.896 | 0.651 | 0.481 | 0.292 | 0.622 | 0.824 | 0.461 | 0.195 | 0.020 |
|  | Δ Getting up | Pearson Correlation | 0.587 | 0.071 | 0.031 | 0.118 | 0.021 | -0.107 | -0.289 | 0.582 | 0.274 | 0.571 |
|  |  | p-value | 0.097 | 0.857 | 0.938 | 0.780 | 0.957 | 0.785 | 0.450 | 0.130 | 0.475 | 0.108 |
|  | Δ Walking | Pearson Correlation | 0.649 | 0.618 | 0.342 | -0.718^*^ | 0.493 | -0.707^*^ | -0.052 | 0.158 | 0.198 | 0.499 |
|  |  | p-value | 0.058 | 0.076 | 0.367 | 0.045 | 0.177 | 0.033 | 0.895 | 0.709 | 0.609 | 0.171 |
|  | Δ Toilet use | Pearson Correlation | 0.806^**^ | 0.247 | -0.082 | -0.380 | 0.345 | -0.334 | -0.275 | 0.536 | 0.586 | 0.678^*^ |
|  |  | p-value | 0.009 | 0.522 | 0.835 | 0.353 | 0.363 | 0.380 | 0.473 | 0.171 | 0.097 | 0.045 |
|  | Δ Grip and hand use | Pearson Correlation | 0.993^**^ | 0.288 | 0.096 | -0.063 | 0.226 | -0.315 | -0.388 | 0.081 | 0.580 | 0.952^**^ |
|  |  | p-value | 0.000 | 0.452 | 0.806 | 0.882 | 0.558 | 0.409 | 0.302 | 0.848 | 0.102 | 0.000 |
|  | Δ Daily activities | Pearson Correlation | 0.712^*^ | 0.428 | 0.053 | -0.632 | 0.116 | -0.317 | -0.553 | 0.022 | 0.403 | 0.517 |
|  |  | p-value | 0.031 | 0.250 | 0.893 | 0.093 | 0.767 | 0.407 | 0.122 | 0.960 | 0.283 | 0.154 |
|  | **. Correlation is significant at the 0.01 level (2-tailed). | | | | | | | | | | | |
|  | *. Correlation is significant at the 0.05 level (2-tailed). | | | | | | | | | | | |

*Abbreviations*: BMI = Body Mass Index; BMD = Bone Mineral Density; FMI = Fat Mass Index; ASMMI = Appendicular Skeletal Muscle Mass Index; BASFI = Bath Ankylosing Spondylitis Functional Index; BASDAI = Bath Ankylosing Spondylitis Disease Activity Index; HAQ = Health Assessment Questionnaire.
